# Enhanced Imaging of Ocular Surface Lesions

**DOI:** 10.3390/jcm15010289

**Published:** 2025-12-30

**Authors:** Wisam O. Najdawi, William R. Herskowitz, Diego E. Alba, Omar Badla, Pragat J. Muthu, Anat Galor, Carol L. Karp

**Affiliations:** 1 Bascom Palmer Eye Institute, University of Miami Miller School of Medicine, Miami, FL 33136, USAagalor@med.miami.edu (A.G.); 2 Department of Ophthalmology, Miami Veterans Administration Medical Center, Miami, FL 33125, USA

**Keywords:** high-resolution optical coherence tomography, AS-OCT, optical coherence tomography angiography, OCTA, ultrasound biomicroscopy, UBM, in vivo confocal microscopy, IVCM

## Abstract

Ocular surface lesions represent a diverse group of pathologies which may be challenging to diagnose clinically. Anterior segment imaging—including anterior segment optical coherence tomography (AS-OCT), optical coherence tomography angiography (OCTA), ultrasound biomicroscopy (UBM), and in vivo confocal microscopy (IVCM)—provides valuable adjunct information for the diagnosis, management, and monitoring of these lesions. The present review aims to provide an update on the principles, current clinical applications, advantages, limitations, and recent advancements in the imaging modalities used for the evaluation of ocular surface lesions. Notable recent advancements include the application of artificial intelligence in the interpretation of AS-OCT, intraoperative use of AS-OCT, the development of three-dimensional UBM, and expanded applications of each modality for a variety of ocular surface lesions.

## 1. Introduction

Ocular surface lesions encompass a diverse range of benign, pre-malignant, and malignant pathologies. While often obvious, they may be subtle, present with similar clinical features, or coexist with other ocular surface diseases [1]. This reality can present a challenge for the diagnosis and monitoring of ocular surface pathologies, even among experienced clinicians. As such, anterior segment imaging modalities are valuable adjuncts to complement clinical examination and histopathological analysis. Recent advancements in these technologies have provided unprecedented levels of detail and insight into these lesions that were only previously attainable with histology.

The purpose of the present review is to provide an update on the principles, current clinical applications, advantages, limitations, and recent advancements in anterior segment imaging modalities used in the evaluation of ocular surface lesions. The imaging modalities discussed include anterior segment optical coherence tomography (AS-OCT), optical coherence tomography angiography (OCTA), ultrasound biomicroscopy (UBM), and in vivo confocal microscopy (IVCM). Of note, while the utility of high-quality clinical photographs, computed tomography (CT), and magnetic resonance imaging (MRI) for the evaluation and monitoring of ocular pathologies cannot be understated, these imaging modalities are beyond the scope of this review and will not be discussed.

## 2. Anterior Segment Optical Coherence Tomography

### 2.1. Background

AS-OCT is a powerful, non-contact imaging modality that can produce high-resolution, cross-sectional images of the anterior structures of the eye, including the cornea, conjunctiva, anterior chamber, and iris [2]. AS-OCT utilizes the principle of low-coherence interferometry, enabling reconstruction of detailed images of the anterior segment. Early devices used the time domain (TD) platform, which uses broadband light and a reference mirror moving in a linear fashion to produce A-scans that are combined to create a single cross-sectional image (B-scan). Although TD AS-OCT provides excellent tissue penetration, the resulting image resolution and acquisition speed are limited (400–2000 A-scans/s) [3,4]. Consequently, there has been a transition to the spectral domain (SD) and swept-source (SS) platforms, both of which utilize Fourier transformation analysis [3]. SD AS-OCT uses fixed broadband light and spectrometers to simultaneously capture numerous A-scans, resulting in higher-resolution images and faster acquisition speed (26,000–70,000 A-scans/s) than TD scans [3]. This platform achieves axial resolutions ranging from 5–7 microns (high-resolution) to 1–5 microns (ultra-high-resolution) [5]. SS AS-OCT uses a sweeping laser source that rapidly changes wavelengths and a dual-balanced photodetector that measures interference signal intensity at each wavelength [6]. This platform achieves greater penetration and faster acquisition speeds (100,000 A-scans/s) than both TD and SD AS-OCT [3,6]. The AS-OCT devices most commonly used in clinical practice at the time of writing employ the SD platform.

AS-OCT provides structural detail of the ocular surface that is valuable in the diagnosis, differentiation, and management of numerous ocular surface lesions [7,8]. Understanding the normal anatomy of the conjunctiva and cornea is essential for identifying landmarks and abnormal scans. On a normal AS-OCT, the most superficial layer of the ocular surface is the tear film, which appears as an ultrathin, hyperreflective band overlying the epithelium. The conjunctival epithelium appears as a thin band of hyporeflective tissue superficial to the subepithelial substantia propria, which appears as a thick, heterogeneous, hyperreflective band [9]. On normal corneal scans, an ultrathin, hyperreflective tear film is seen superficially, followed by a thin, hyporeflective epithelium, a thick, variably reflective stroma, and lastly a thin, hyperreflective endothelium in the central cornea. As scans approach the corneoscleral limbus, the epithelium becomes slightly more hyperreflective and the subepithelial tissue becomes slightly more disorganized and hyperreflective [9].

### 2.2. Current Applications of AS-OCT

High-resolution AS-OCT has become a helpful tool for the diagnosis and treatment of several ocular pathologies, including keratoconus [10], corneal ulcers [11], and narrow angle glaucoma [12]. More recently, HR-OCT has also been used in the diagnosis, differentiation, and management of various ocular surface lesions. The unique features of these lesions in AS-OCT allow distinction between pathologies, especially in clinically similar-appearing benign and malignant lesions. Differentiating between benign and malignant lesions is essential to ensuring prompt management and favorable outcomes.

**Figure 1:**
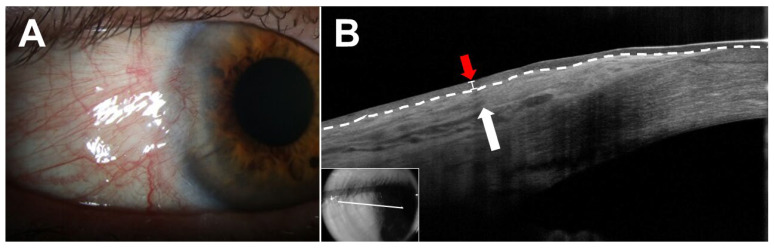
Pterygium. (**A**) Slit lamp examination of the right eye of a 71-year-old Hispanic male revealed a fibrovascular perilimbal lesion from 8:00 to 10:00. (**B**) Anterior segment optical coherence tomography (AS-OCT) demonstrated normal thickness epithelium and a hyperreflective subepithelial lesion (white arrow) with a “stringy” or fibrous appearance. The border between the epithelium and subepithelial tissue is demarcated by a white dashed line. Epithelial thickness over the lesion was measured to be approximately 60 microns (white ladder adjacent to red arrow). Inset shows raster location.

Among benign lesions, several pathologies may mimic ocular surface squamous neoplasia (OSSN), including pterygia, pinguecula, and conjunctival papillomas. Pterygia are benign, subepithelial fibrovascular growths of the ocular surface that often masquerade as OSSN [13,14,15]. Additionally, they may harbor occult or unexpected OSSN in up to 15% of cases [16]. AS-OCT can reliably differentiate between these lesions. Pterygia appear as hyperreflective subepithelial lesions with a “stringy” or fibrous appearance and demonstrate normal thickness epithelium in AS-OCT (Figure 1). As such, several studies have evaluated epithelial thickness to distinguish OSSN from pterygia. With epithelial cutoff thicknesses in the range of 97–142 microns, AS-OCT demonstrates excellent sensitivity (range: 94.0–100) and specificity (range: 94.7–100) for the differentiation of OSSN and pterygia using various AS-OCT systems [9,17,18,19]. AS-OCT can also be used to differentiate OSSN from papillomas, benign epithelial lesions. Although papillomas also present with a thickened, hyperreflective epithelium, the epithelium often exhibits a characteristic “mushrooming” over the normal epithelium in contrast to the abrupt transition seen in OSSN [20]. Additionally, papillomas frequently demonstrate epithelial invaginations/corrugations and intrinsic spaces, which are generally absent in OSSN [20]. Gündüz et al. reported a median maximal epithelial thickness of 560 microns and 965.5 microns in OSSN and papillomas, respectively, in AS-OCT [21]. Using an epithelial thickness cutoff of 630.5 microns, AS-OCT was able to differentiate OSSN from conjunctival papillomas with 69% sensitivity and 100% specificity [21].

AS-OCT can also be beneficial in the evaluation of the pigmented precursor lesions of conjunctival melanoma (CM). As with OSSN, CM may clinically resemble precursor lesions such as conjunctival nevi and primary acquired melanosis (PAM). AS-OCT can help to effectively distinguish these entities [22]. In contrast to CM, conjunctival nevi typically present as well-circumscribed subepithelial lesions containing intralesional cysts and normal overlying epithelium [8,23]. In contrast, PAM demonstrates a band of basal epithelial hyperreflectivity with no subepithelial involvement [22]. However, limitations exist as AS-OCT cannot distinguish between PAM with and without atypia.

AS-OCT has also been applied to the characterization of several other benign subepithelial lesions, including benign reactive lymphoid hyperplasia (BRLH) [24], conjunctival myxoma [25,26], conjunctival neuroma [27,28], conjunctival amyloidosis [24], and blue nevus [29].

**Figure 2:**
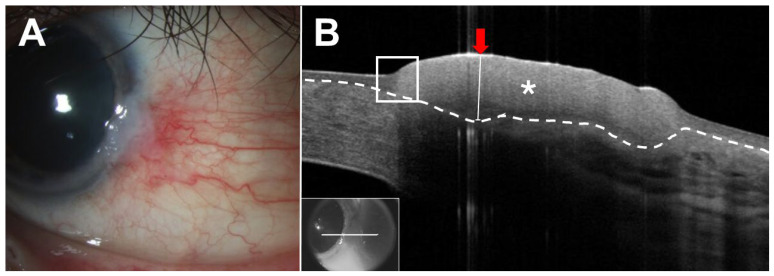
Ocular surface squamous neoplasia. (**A**) Slit lamp examination of the left eye of a 60-year-old Hispanic male revealed a gelatinous perilimbal lesion from 2:30 to 4:00. (**B**) Anterior segment optical coherence tomography (AS-OCT) demonstrated a hyperreflective epithelial lesion (asterisk) with an abrupt transition from normal to abnormal (white box). The border between the epithelium and subepithelial tissue is demarcated by a white dashed line. Epithelial thickness through the thickest part of the lesion was measured at approximately 452 microns (white ladder adjacent to red arrow). Inset shows raster location.

Regarding malignant lesions, several studies have described the appearance of OSSN, a spectrum of epithelial malignancies affecting the cornea and conjunctiva, in AS-OCT. Classically, these lesions demonstrate a thickened, hyperreflective epithelium and an abrupt transition from normal to abnormal tissue (Figure 2) [9,30,31,32]. Together, these features have shown high sensitivity and specificity for the diagnosis of OSSN [7,9,31,32,33,34]. However, not all images demonstrate all three features and, as such, it is important to examine all cuts through a lesion to look for characteristic abnormalities. Importantly, the absence of one or more of these features does not rule out a diagnosis of OSSN, and clinicians must correlate AS-OCT findings with clinical findings and histopathological assessment. OSSN may occur in the setting of coexisting inflammation or scarring, such as in patients with ocular cicatricial pemphigoid, herpes simplex virus keratitis, atopy, or limbal stem cell deficiency [35]. Identifying the classic epithelial features of OSSN enables clinicians to detect malignancy even in the setting of coexisting ocular surface disease [36]. In a study of 16 patients with concurrent ocular surface abnormalities who underwent biopsy, AS-OCT correctly identified OSSN in 12 cases and ruled it out in 4 cases in clinically ambiguous lesions [36]. Notably, the features of nodulo-ulcerative OSSN (invasive squamous cell carcinoma; SCC), a rare and aggressive clinical subtype, differ from classic OSSN [37]. In a series of 16 cases, in addition to the classic features of OSSN, nodulo-ulcerative OSSN demonstrated epithelial thinning and melt in some areas rather than thickening [37]. AS-OCT demonstrated limbal, corneal, and scleral thinning in many cases [37]. Additionally, the presence of a “wedge sign”, a triangular corneal stromal opacity with its base facing the lesion separated from the intact epithelium by normal stroma, indicated stromal tumor invasion with 100% specificity and positive predictive value [37]. Of note, though exceedingly rare, conjunctival basal cell carcinoma may mimic OSSN in AS-OCT. These lesions demonstrate a thickened, hyperreflective epithelium with an angled transition between normal and abnormal tissue [38]. Importantly in all pathologies, some images are easier to analyze than others, and the presence of coexisting disease may make images more difficult to interpret.

**Figure 3:**
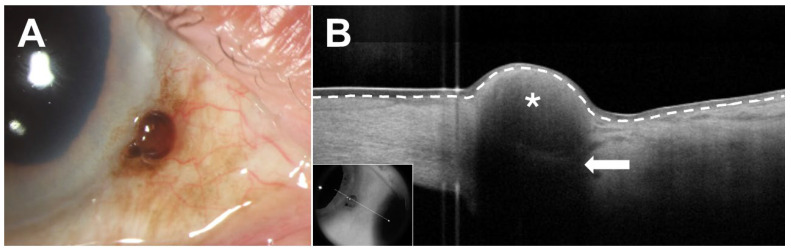
Conjunctival melanoma (CM). (**A**) Slit lamp examination of the right eye of an 86-year-old White male revealed a focal elevated, pigmented lesion at the corneoscleral limbus from 3:30 to 4:30 with adjacent corneal and conjunctival melanosis from 3:00 to 5:00. Biopsy confirmed a 600-micron CM with adjacent primary acquired melanosis. The deep and peripheral conjunctival margins were clear. (**B**) Anterior segment optical coherence tomography (AS-OCT) demonstrated an elevated, hyperreflective subepithelial lesion (asterisk) with normal thickness, slightly hyperreflective overlying epithelium, and mild posterior shadowing (arrow). The border between the epithelium and subepithelial tissue is demarcated by a white dashed line. Inset shows raster location.

AS-OCT of CM typically reveals an elevated, hyperreflective, subepithelial lesion with normal thickness epithelium, variably hyperreflective epithelium, and significant posterior shadowing in larger lesions (Figure 3) [5,9,39,40]. Differentiating CM and its precursor lesions is essential for ensuring appropriate management. While conjunctival nevi and PAM with low to moderate atypia can often be monitored, PAM with severe atypia and CM typically require more invasive treatment, such as excisional biopsy with adjuvant cryotherapy or topical chemotherapy [41,42,43]. As noted above, however, AS-OCT images do not differentiate between PAM with mild versus severe atypia and, as such, clinical judgment and consideration of biopsy is paramount to developing an appropriate treatment plan.

**Figure 4:**
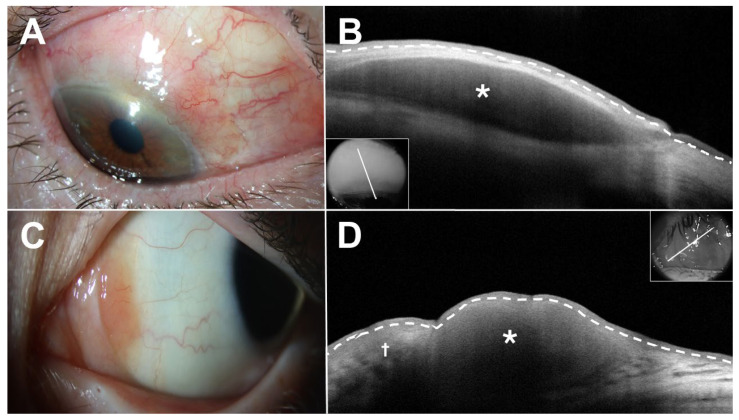
Conjunctival lymphoma (**A**,**B**) versus benign reactive lymphoid hyperplasia (**C**,**D**). (**A**) Slit lamp examination of the right eye of a 76-year-old White male revealed a subtle salmon-colored conjunctival lesion from 10:00 to 3:00. (**B**) Anterior segment optical coherence tomography (AS-OCT) demonstrated a homogeneous subepithelial hyporeflective lesion (asterisk) with dot-like infiltrates. Notably, the overlying epithelium was normal thickness and slightly hyperreflective. (**C**) Slit lamp examination of the left eye of a 14-year-old White male revealed a salmon-colored conjunctival lesion nasally adjacent to the nasal canthus. (**D**) AS-OCT demonstrated a somewhat hyporeflective subepithelial lesion (asterisk) with dot-like infiltrates. Notably, the overlying epithelium was normal thickness and slightly hyperreflective. Note the plical tissue at the nasal edge of the lesion (cross). In the AS-OCT images, the border between the epithelium and subepithelial tissue is demarcated by a white dashed line. Inset shows raster location.

AS-OCT has also been applied to the characterization of conjunctival lymphoma (CL), another malignant subepithelial tumor. CLs typically appear as a homogeneous, hyporeflective subepithelial lesion with normal overlying epithelium, smooth borders, and monomorphic dot-like infiltrates (Figure 4) [24]. These lesions may be bordered by a hyperreflective band of substantia propria representing the tissue displaced by the lymphocytic infiltrate corresponding with histopathologic specimens. While CL and BRLH may appear similar in AS-OCT, CL tends to be more homogenous and better defined [24]. AS-OCT alone is not sufficient for differentiating CL from BRLH, and a biopsy is essential for confirming the diagnosis and developing a treatment plan.

### 2.3. Advantages and Disadvantages

AS-OCT is a valuable, non-invasive diagnostic modality for evaluating ocular surface lesions. As described, AS-OCT generates high-resolution, cross-sectional images of the ocular surface that allow for detailed structural assessment, accurate differentiation of epithelial and subepithelial lesions, and precise measurement of lesion dimensions. These images can help to guide biopsies and facilitate surgical planning. This is particularly important for atypical lesions that may mimic one another, such as pigmented OSSN, amelanotic nevi, and CM [44,45]. AS-OCT can be a valuable tool in these situations. AS-OCT has also become a useful tool for monitoring treatment response in ocular surface lesions. Lesions treated with excisional biopsy, plaque brachytherapy, or topical chemotherapy can be monitored for treatment response and subclinical recurrence [40,46]. AS-OCT can be a helpful partner when treating OSSN patients medically. In such situations, the OCT can aid in determining when the lesion is resolved and prevent premature termination of treatment. In a study of 95 patients with OSSN treated with topical 5-fluorouracil or interferon-alpha-2b, AS-OCT detected the persistence of subclinical disease despite appearance of clinical resolution in 17% of cases [46]. Other key benefits of AS-OCT are its ease of use and rapid learnability for novice clinicians. One study demonstrated that clinicians with limited prior experience, including ophthalmology residents, fellows, and optometry students, significantly improved their diagnostic accuracy for OSSN after a 20 min teaching session [47]. Among 34 clinicians, diagnostic accuracy for distinguishing OSSN from non-OSSN lesions improved from 70% to 84% following this brief training. This suggests that with focused training, clinicians can utilize this technology.

Despite its many advantages, AS-OCT has several limitations. Notably, while some lesions demonstrate the classic AS-OCT features described above, some images may not contain all notable features, have poor image quality, or be more difficult to interpret. As such, it is important to capture several images throughout the lesion and correlate clinically when interpreting AS-OCT scans. Additionally, AS-OCT lacks the resolution needed to assess the cellular-level details required to identify atypia. For example, while AS-OCT can identify basal hyperreflectivity corresponding to melanocytic pigmentation in PAM, it cannot distinguish between lesions with and without atypia [22]. As such, histopathologic analysis remains necessary for definitive diagnosis. Another limitation of AS-OCT is difficulty visualizing the posterior margins of thick or pigmented lesions due to obscuration from posterior shadowing [48,49]. A systematic review comparing AS-OCT with UBM found that the decreased scanning depth of 1–3 mm for AS-OCT was more effective for small lesions, whereas UBM provided better resolution of the posterior margin in deep and pigmented lesions [50].

### 2.4. Recent Advances

In recent years, advances in AS-OCT technology for ocular surface lesions have expanded its potential utility in the clinic and operating room, though it is not yet part of routine clinical practice. Artificial intelligence (AI) has emerged as a means to enhance the utility of AS-OCT by enabling automated, accurate image interpretation, even in difficult-to-interpret images or images lacking classic features. AS-OCT AI has already been applied to several anterior segment pathologies, including keratoconus [51], angle-closure glaucoma [52], and, more recently, ocular surface lesions [53]. In a 2025 study, a deep learning model was developed to differentiate OSSN from benign lesions of pterygia and pinguecula using AS-OCT. The AI model achieved excellent performance, with a sensitivity of 86.4% and specificity of 93.2%, surpassing clinicians in sensitivity (69.8%) and maintaining comparable specificity (98.5%) [53]. These findings suggest that AS-OCT AI could aid in screening for ocular surface lesions, especially in settings lacking subspecialty expertise. The long-term objective is to embed this software within the machine, enabling clinicians to obtain interpretation guidance alongside the images. In addition to assisting with diagnosis, multiple groups have applied deep learning networks to define tissue borders in AS-OCT images [54,55]. Future segmentation algorithms may prove valuable for the evaluation of ocular surface lesions. AS-OCT has also shown early promise in guiding the surgical management of ocular surface lesions. In a pilot study, AS-OCT was used to map tumors preoperatively in eight patients undergoing OSSN excision, akin to an “optical Mohs” [56]. The tumor margins predicted by AS-OCT were successfully transferred intraoperatively using anatomical landmarks, and tumor margins were assessed on histopathology. In all eight cases, AS-OCT accurately predicted the true tumor borders, suggesting a potential role in pre- and intraoperative surgical planning [56]. In the future, high-resolution AS-OCT devices may be built into operating microscopes, so clinicians can use the images to help guide surgery. Another recent advancement in AS-OCT involves reconstruction of the transverse sections of B-scans to generate coronal “C-scans”, providing a rapid method of visualizing lesions en face [57,58]. Though the clinical utility of this technology has not yet been established, both Tahiri Joutei Hassani et al. and Bunod et al. have recently applied this technique to evaluate pterygia and OSSN, demonstrating clear visualization of lesion extension and borders [57,58]. Recently, three-dimensional AS-OCT (3D-AS-OCT) has been applied to ocular surface diseases such as Mooren’s ulcer [59]. This technique has not yet been applied to ocular surface lesions but may serve as a valuable diagnostic tool for their evaluation.

### 2.5. Summary

AS-OCT is an important imaging modality that provides high-resolution, cross-sectional images of the ocular surface and anterior segment. It has proven particularly valuable in evaluating ocular surface lesions, allowing for reliable morphological differentiation between benign and malignant entities. Its ability to help clinicians accurately identify lesions, distinguish epithelial from subepithelial involvement, guide biopsy location, and monitor treatment response makes it a vital tool in routine clinical decision-making.

## 3. Optical Coherence Tomography Angiography

### 3.1. Background

OCTA is a non-invasive imaging modality that can rapidly provide detailed morphologic images of ocular vasculature [60]. Image acquisition relies on signals from moving structures within the blood vessels such as red blood cells (RBCs). Successive B-scans are taken at the same region, and the changing signals from the contrast motion of the RBCs generate an image of the vasculature [61]. These images are capable of achieving axial resolutions of 5 microns within a 3 × 3 mm^2^ area [62]. OCTA was initially designed to image the retinal and choroidal vasculature, offering clinical value in conditions such as diabetic retinopathy, retinal venous and arterial occlusions, and macular degeneration, among others [61,63]. As such, most anterior segment OCTA machines use an adaptor lens to account for the anatomical differences between the anterior and posterior segments, while other systems manually adjust the parameters. Currently, there is no standard protocol for OCTA image acquisition or use [64].

### 3.2. Current Applications of OCTA

Though primarily used in posterior segment imaging, OCTA has demonstrated early potential utility in the evaluation of anterior segment pathologies, including ocular surface lesions, although it is not routinely used in ocular surface clinics at this time. Early applications of this technology have generally been limited to the characterization of various ocular surface lesions; however, recent studies have applied OCTA to the differentiation of benign and malignant lesions and monitoring of treatment response.

Among benign lesions, OCTA has been used to analyze conjunctival blood vessel density in pinguecula and pterygia versus healthy eyes [65]. Conjunctival blood vessel density is significantly higher in pterygium eyes compared with pinguecula and normal eyes, which show similar densities [65]. These findings reinforce the roles of angiogenesis and neovascularization in pterygium formation and, as we learn more, may offer diagnostic value [65]. OCTA has also shown utility in characterizing and monitoring conjunctival and graft reperfusion following pterygium surgery, allowing detection of graft hypoperfusion and ischemia earlier than slit lamp examination and enabling early graft exchange [66]. Measurement of graft reperfusion may provide a useful metric to help predict pterygium recurrence in the future, as recurrence has been linked to inadequate graft reperfusion [67].

OCTA has also been used in the characterization of conjunctival nevi and PAM [68]. The blood vessels identified in conjunctival nevi were tortuous, whereas the vessels in PAM were similar to those of a normal conjunctiva. Tomoda et al. used OCTA to describe an inflamed juvenile conjunctival nevus, which appeared as a cystic structure with poor internal vascular signals and abundant underlying scleral vessels [69]. Although studies to date have been largely descriptive and limited by small sample sizes, scaling to larger cohorts may yield pathophysiological understanding or novel diagnostic and prognostic insights.

**Figure 5:**
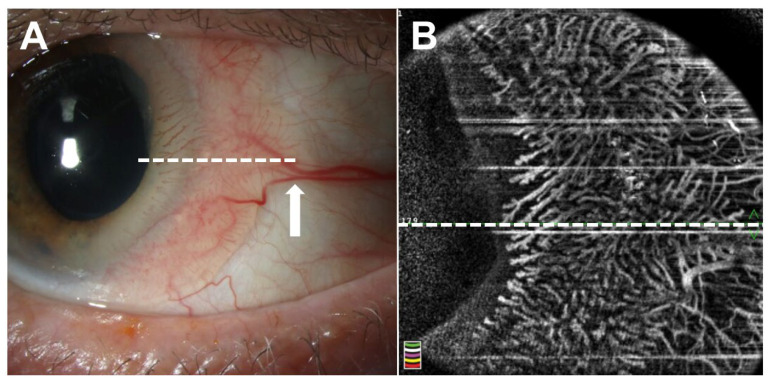
Ocular surface squamous neoplasia. (**A**) Slit lamp examination of the left eye of a 74-year-old White female revealed a gelatinous perilimbal conjunctival lesion from 1:00 to 5:30 with extensive intrinsic vascularity and two large feeder vessels (arrow). (**B**) Optical coherence tomography angiography (OCTA) through the lesion demonstrated a high density of abnormally branching blood vessels (vessel area density [VAD] = 28.79%) in a “sea fan” arrangement. The location of the OCTA image is denoted by a white dashed line in both images.

Regarding malignant lesions, OCTA has been used to characterize the vascular changes in OSSN by studying the vessel area density (VAD) [70] (Figure 5). This study revealed that VAD was highest in the subepithelial tissue beneath the tumor, followed by the tumor itself, and lowest in the adjacent tissue. Additionally, the VAD in the area adjacent to the tumor was higher than in the corresponding region of the uninvolved eye. [70]. OCTA has also been used to monitor vascular changes following OSSN treatment with topical chemotherapy and immunotherapy [71,72]. Treatment resulted in reduction and normalization of the VAD, both within the tumor and the subepithelial tissue below, with tumor resolution [72]. Qualitatively, intraepithelial vascular networks, present prior to treatment, disappeared and subepithelial vessels appeared less tortuous and more organized as treatment progressed [72]. Interestingly, when evaluating patients during their treatment with topical 5-fluorouracil, the mean VAD in the subepithelium adjacent to the OSSN increased early in treatment, then decreased significantly between mid-treatment and resolution. This may explain why patients sometime appear worse before they look better when on topical treatment. By understanding blood vessel patterns within tumors and tracking how they change with therapy, we can gain valuable insights into tumor physiology and treatment mechanisms. Larger datasets and quantitative analyses are still needed.

A recent report used OCTA to compare the vascular patterns of pterygia and OSSN. This study reported zigzag vessels in OSSN versus straight vessels in pterygia, a feature that may serve to distinguish the two pathologies in ambiguous cases [73]. Among melanocytic malignancies, OCTA has been used to characterize CM, identifying tortuous vasculature compared with the adjacent vessels [68]. Additionally, Kiseleva et al. identified an increase in perfusion density and uneven blood vessel caliber in CM versus conjunctival nevi, which may aid diagnosis in ambiguous lesions [74].

### 3.3. Advantages and Disadvantages

OCTA is a rapid, non-invasive, safe, and repeatable imaging modality that can detect small-caliber vessels, making it an important tool in research [64]. OCTA offers imaging resolution comparable with that of indocyanine green angiography (ICGA) and fluorescein angiography (FA), without the risks associated with contrast administration. In addition, since intravenous dye is not required, imaging can be performed by a technician without the need for physician oversight.

Despite these advantages, several limitations affect the utility of OCTA for ocular surface lesions. First, while the uses of OCTA for the posterior segment are well established, there remains a paucity of literature discussing the applications of OCTA for anterior segment pathologies. As such, at the time of this writing, not enough information is available to determine how OCTA can be used in the diagnosis and management of ocular surface lesions. Additionally, the observations in studies using OCTA to date are largely qualitative, so further investigation is required to identify quantitative measures by which to evaluate lesions. Regarding image acquisition, image quality may be affected by motion and projection artifacts. Additionally, the technique is operator-dependent, so image quality may vary with technician experience. Since imaging relies solely on the movement of intravascular structures, OCTA is unable to distinguish arteries from veins or identify areas of leakage. Furthermore, OCTA demonstrates limited penetration through opaque tissues, such as with hyperkeratotic lesions or scarring, and it provides a relatively limited field of view.

### 3.4. Recent Advances

The use of OCTA to diagnose and differentiate benign versus malignant ocular surface lesions is very limited at the time of this writing. In 2021, Binotti et al. identified perilesional blood vessel depth and diameter as metrics capable of differentiating benign epithelial lesions (pterygium and pingueculae), pigmented lesions (conjunctival nevi and complexion-associated melanosis), and lymphoid lesions (BRLH, conjunctival granulomas, and conjunctival inclusion cysts) from their malignant counterparts (OSSN, PAM with severe atypia/melanoma in situ or CM, and conjunctival lymphoma, respectively) [75]. Malignant lesions demonstrated deeper perilesional vessels than benign lesions, with a sensitivity of 90.9%, specificity of 100.0%, and area under the curve (AUC) of 0.980 for identifying malignancy at a cutoff of 236.5 microns [75]. Additionally, malignant lesions demonstrated greater perilesional vessel diameter than benign lesions, with a sensitivity of 100.0%, specificity of 88.9%, and AUC of 0.960 for identifying malignancy at a cutoff of 53.9 microns [75]. This study shows that vessel information could serve as a useful adjunct for detecting malignant lesions in the future. Recently, ultra-widefield OCTA has been applied to the retina [76]. This technique has not yet been applied to ocular surface lesions but may improve the utility of OCTA for their evaluation.

### 3.5. Summary

OCTA is a relatively new, non-invasive imaging modality that visualizes ocular vasculature without the need for contrast dyes. While it has established applications in the posterior segment, its role in anterior segment imaging remains largely investigational. Recent studies suggest that OCTA may aid in characterizing vascular patterns of ocular surface lesions and differentiating benign from malignant lesions; however, OCTA is not routinely used in clinical practice for ocular surface lesions due to limited evidence, lack of standardized protocols, and limited availability.

## 4. Ultrasound Biomicroscopy

### 4.1. Background

UBM is a useful imaging modality in the evaluation and management of many ocular lesions. This technique, developed in 1990, uses high-frequency ultrasonography (35–100 MHz) to dynamically visualize ocular structures and lesions [77,78]. A transducer emits ultrasonic waves which reflect off tissue interfaces, and the reflected waves are detected by the transducer to be converted into an image based on time delay and signal intensity. While its resolution is lower than that of other imaging modalities, it provides excellent tissue penetration with minimal shadowing in real time. Signal penetration depends on the frequency used and the ultrasound attenuation coefficient of the tissue being imaged [79]. Using lower frequencies allows deeper visualization and wider fields of view, while higher frequencies have greater resolution [80]. At a frequency of 50 MHz, UBM provides approximately 25 microns of axial resolution and 50 microns of lateral resolution to a depth of roughly 5 mm [77].

### 4.2. Current Applications of UBM

Originally described for use in ocular surface lesions in 1994, UBM is particularly beneficial in the evaluation of tumor margins and intraocular extension [81]. Since then, UBM has been applied to a variety of ocular surface lesions, including benign and malignant pathologies. However, given its relatively poor resolution, UBM does not excel in diagnosing or differentiating pathologies. As such, the primary role of UBM, detecting scleral and intraocular invasion, has remained relatively unchanged since its inception.

Among benign lesions, UBM has been used to evaluate conjunctival nevi, which demonstrate low internal echogenicity, are located deep to the conjunctiva, and contain numerous anechoic cysts [82]. In a series of 57 conjunctival nevi, UBM was able to identify the posterior border of nevi in 100% of cases, even in heavily pigmented or thick lesions; however, its ability to detect intralesional cysts was limited, with a detection rate of only 28.5% [48]. Notably, UBM cannot distinguish between conjunctival nevi and CM arising from nevi as both lesions may contain intralesional cysts and UBM cannot provide cellular detail [82]. UBM has also been applied to the evaluation of conjunctival myxomas, which demonstrate dome-shaped subepithelial lesions with homogenous low to medium internal echogenicity, numerous hypoechogenic vascular foci, and well-defined margins [25,83,84]. In the case of a rare primary corneal myxoma, UBM was able to reliably discern that the lesion had not violated Bowman’s layer [83]. In addition, UBM has been used to characterize various benign nerve sheath tumors. Schwannomas typically demonstrate low internal echogenicity [85], whereas neurofibromas appear solid, homogeneous, and exhibit medium to high echogenicity [86]. Additionally, UBM has been used to evaluate caruncular oncocytomas, which demonstrate mixed solid and cystic components, moderate internal reflectivity, and poor demarcation between the lesion and underlying tissue [87,88].

**Figure 6:**
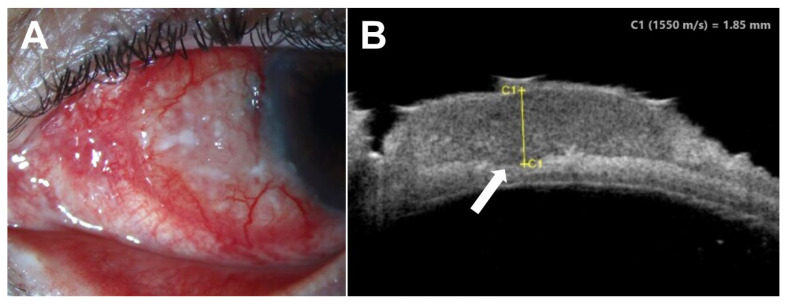
Invasive squamous cell carcinoma of the conjunctiva. (**A**) Slit lamp examination of the left eye of a 53-year-old Hispanic female revealed an elevated, gelatinous conjunctival lesion from 7:00 to 10:30. (**B**) Ultrasound biomicroscopy (UBM) demonstrated a 1.85 mm thick, hypoechoic epithelial lesion with evidence of subepithelial/scleral invasion (arrow). Notably, the resolution of UBM does not provide significant intratumoral detail as with anterior segment optical coherence tomography or histopathology, so these diagnostic modalities should be used in tandem.

Regarding malignant lesions, UBM has been applied extensively to the evaluation of OSSN, particularly to determine if lesions have progressed to invasive SCC (Figure 6). In UBM, OSSN demonstrates a highly hyperechoic surface with a hypoechoic stroma [89]. Tumors may contain hyperechoic foci consistent with keratin accumulation [90]. Features suggestive of intraocular invasion include diminished stromal reflectivity underlying the tumor, a visible anterior segment mass, uveal thickening, or blunting of the iridocorneal angle [89,90,91,92]. Additional applications of UBM in the management of OSSN include identification of tumor margins prior to surgical excision [93] or serial measurement of tumor dimensions to monitor response to non-surgical interventions [89].

UBM can also be used for CMs, which appear as dome-shaped, subepithelial lesions with a slightly hypoechoic core in UBM [40]. In CM, multiple studies encompassing 957 total patients showed that between 70 and 89% of tumors demonstrate some degree of pigmentation [94,95,96]. While AS-OCT is better suited to differentiating CM from its precursor lesions, heavy pigmentation may obscure intratumoral details or lead to posterior shadowing. Therefore, UBM is preferred for measurement of tumor dimensions and identification of intraorbital extension in CM. UBM has been reported to reliably measure tumor thickness within 0.0–0.5 mm of the thickness measured on histopathology [97]. Additionally, UBM may aid in differentiating intraocular invasion of CM versus extraocular extension of uveal melanoma [98]. UBM has been used to measure scleral thickness following surgical excision of CM with cryotherapy [99].

The use of UBM for CL has been limited. One case report was identified that described CL in UBM. In this report, the CL demonstrated a hypoechoic, subepithelial lesion with hyperechoic septations that were mobile relative to the underlying sclera [100]. Additionally, as with other large ocular lesions, UBM allows for visualization of the posterior margin of large CLs [40]. Lastly, a single case report was identified that described the appearance of conjunctival myeloid sarcoma in UBM. This lesion appears hyperechogenic with linear thickening and is adherent to the underlying tissue [101].

### 4.3. Advantages and Disadvantages

UBM is a low-cost ancillary imaging modality that provides real-time, cross-sectional images of the ocular surface and anterior segment. It offers excellent depth of penetration, enabling precise measurement of lesion dimensions and identification of posterior tumor margins. Because UBM relies on ultrasonic waves rather than light, its imaging capability is largely unimpeded by thick or heavily pigmented lesions.

Despite its advantages, UBM has several notable limitations. Its image resolution is inferior to that of other imaging modalities, making it difficult to appreciate fine intralesional details. Additionally, image acquisition requires direct contact of the transducer with the eye using coupling media in an immersion bath or acoustically transparent film bag, which may cause patient discomfort [79]. In addition, imaging requires patients to lie supine, which may be challenging for patients with certain medical comorbidities. Last, the modality requires a skilled operator, especially when acquiring high-resolution UBM scans, and thus image quality can be highly operator-dependent.

### 4.4. Recent Advances

The primary application of UBM in the evaluation and monitoring of ocular surface lesions is to detect scleral and intraocular invasion. However, an interesting advancement involves the development of a custom-built operating microscope-mounted UBM system that enables the acquisition of three-dimensional (3D) ultrasound images of the anterior segment [102]. Though not yet commercially available, this technology will allow detailed and reproducible measurement of tumor volume, which may be important for treatment planning for ocular surface lesions. Additionally, the described 3D UBM automates the image acquisition process, reducing reliance on operator skill. Future applications may include adaptation of this technology for use outside of the operating room. In addition to technological advances, UBM was recently used to evaluate a basal cell carcinoma for scleral invasion [103].

### 4.5. Summary

UBM is a valuable imaging technique that provides real-time, cross-sectional imaging of the anterior segment with excellent tissue penetration. Although its resolution is lower than that of AS-OCT, UBM, especially high-resolution modalities, excel in identifying tumor borders, even in thick or pigmented lesions where light-based imaging is limited. Clinically, UBM is widely used in the evaluation of ocular surface tumors and is an essential tool in routine practice.

## 5. In Vivo Confocal Microscopy

### 5.1. Background

IVCM is another imaging modality that typically requires contact and provides high-resolution, en face images of the ocular surface [104,105,106]. Of note, ConfoScan (Nidek Inc., Colony, TX, USA) was previously available as a non-contact option for IVCM, but at the time of writing, this model is no longer commercially available. Originally developed in 1955 [107], IVCM functions by focusing a point light source and detection optic onto the same focal plane. The detection optic is positioned behind a small aperture which restricts scattered light from out-of-focus planes from reaching the photodetector, thereby reducing background noise and enhancing image resolution [104,105,106]. Since resulting images represent only a small plane within the specimen, multiple scans are required to evaluate the tissue architecture [104,105,106]. IVCM achieves resolutions of between 0.2–4.0 microns laterally and 0.6–4.0 microns axially [105,108] and enables visualization up to a depth of approximately 130 microns, roughly the depth of the superficial substantia propria [109]. The most common IVCMs used in clinical practice are laser scanning confocal microscopes (LSCMs), which utilize a laser light source and galvanometer mirrors to rapidly capture images [105].

### 5.2. Current Applications of IVCM

Given its ability to non-invasively capture detailed images at the cellular level, IVCM has been employed in the investigation of various ocular surface pathologies, though its clinical utility is limited. Historically, its primary applications have included the assessment of infectious keratitis, especially the detection of acanthamoeba and fungi [110,111,112], dry eye disease [113,114,115], inflammation [116,117], and corneal nerves [118,119,120,121]. Less commonly, it has also been applied to benign and malignant ocular surface lesions.

Among benign and pre-malignant lesions, pterygia have been characterized in IVCM by decreased corneal epithelial cell density, increased epithelial cell size, reduced nucleus-to-cytoplasm ratio, sharp cellular borders, an increased number of dendritic cells, shorter corneal nerve fiber length, and a hyperreflective stroma [122,123,124,125]. However, one study of 104 patients reported no significant differences in epithelial or corneal nerve cell densities compared with healthy controls [126]. Papillomas demonstrate loss of normal epithelial architecture with variable-sized hyperreflective cells in IVCM [127]. Conjunctival amyloidosis presents with deposits of acellular, hyporeflective lobules adjacent to blood vessels within the substantia propria [128]. Few case reports have described conjunctival neuromas using IVCM. These lesions consist of disorganized bundles of enlarged subconjunctival, hyperreflective nerves with dilations, loops, bifurcations, and hyporeflective borders [129,130,131].

IVCM has also been used in the study of several pigmented ocular surface lesions. Conjunctival nevi demonstrate clusters of round, medium-sized subepithelial cells that are hyporeflective centrally with hyperreflective borders and large pseudocysts lined with stratified, nonkeratinized epithelium and filled with monomorphic material [122,132,133]. PAM exhibits hyperreflective cells and granules along the basal conjunctival epithelium with otherwise normal epithelium [122,133]. In the presence of atypia, large arborizing networks of hyperreflective dendritic cells may be present [133].

**Figure 7:**
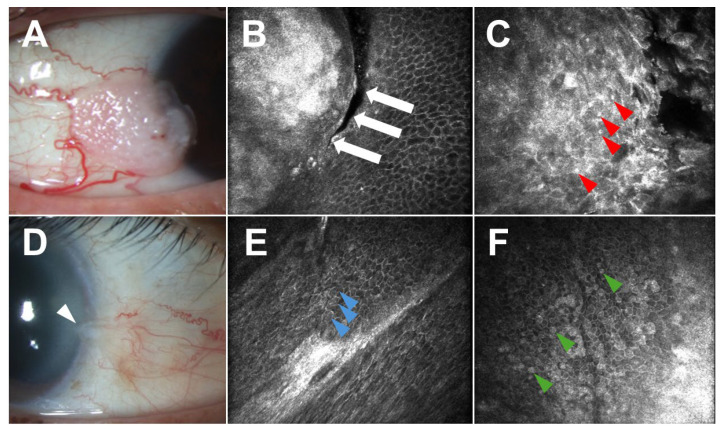
Ocular surface squamous neoplasia (OSSN; (**A**–**C**)) versus squamous metaplasia (**D**–**F**). (**A**) Slit lamp examination of the right eye of a 55-year-old White male revealed a papillary perilimbal lesion from 8:00 to 10:00 with two large feeder vessels inferotemporally. (**B**) In vivo confocal microscopy (IVCM) demonstrated an abrupt transition (white arrows) between normal and abnormally enlarged, pleomorphic, and hyperreflective squamous epithelial cells. (**C**) IVCM also demonstrated highly pleomorphic, keratinized abnormal epithelial cells with numerous mitotic figures (red arrowheads), hyperreflective nuclei, and increased nuclear–cytoplasmic ratios in this biopsy-proven OSSN. (**D**) Slit lamp examination of the right eye of a 75-year-old Hispanic female revealed a subtle opalescent conjunctival lesion (white arrowhead) from 3:00 to 4:00, which stained with lissamine green. Biopsy revealed foci of squamous metaplasia without malignancy. (**E**) IVCM demonstrated an abnormal cluster of metaplastic epithelial cells (blue arrowheads). (**F**) IVCM also demonstrated metaplastic hyperreflective epithelial cells (green arrowheads) interspersed among normal epithelial cells. Notably, while the cellular features identified in IVCM may be consistent with OSSN, this modality lacks the sensitivity and specificity to definitively distinguish malignant and benign lesions. Therefore, IVCM should be obtained in conjunction with histopathology.

Regarding malignant lesions, numerous studies have characterized OSSN using IVCM. These lesions appear hyperreflective with enlarged, pleomorphic epithelial cells; bright, variably sized nuclei; high nuclear-to-cytoplasmic ratios; actively mitotic cells; and a clear distinction between normal and neoplastic epithelium (Figure 7) [134,135,136,137,138]. In some cases, large fibrovascular structures and/or fine blood vessels may be visible within the lesion [139]. While IVCM can reliably differentiate OSSN from normal tissue, it demonstrates poor sensitivity and specificity for distinguishing between benign lesions, including pterygia, pinguecula, and papillomas, and OSSN (38.5% and 66.7%, respectively) [140]. Few studies have described the appearance of CM using IVCM, demonstrating subepithelial accumulations of large cells with hyperreflective nuclei, prominent hyporeflective nucleoli, surrounding hyperreflective inflammatory cells, and large intralesional blood vessels [122,133]. CL is characterized by a combination of anucleate, polygonal cells with obscured borders and round, hyperreflective cells in the subepithelial space. These cells are distributed diffusely and arranged in hyporeflective cystic spaces [141].

### 5.3. Advantages and Disadvantages

IVCM is an adjunct imaging modality that has sparingly been used in the evaluation and monitoring of ocular surface lesions. Its strengths are that it is non-invasive (but requires contact), enables extremely high-resolution visualization at the cellular level, and can be performed in real time. Additionally, IVCM allows for quantitative assessment of cellular density, cellular morphology, and the presence of inflammatory cells.

Despite these advantages, there are several notable disadvantages that limit the clinical utility of IVCM. First, IVCM provides only a small, single-plane field of view of the tissue of interest, making imaging large or thick lesions challenging and time-consuming. Even in the case of small lesions, imaging may take 5–15 min and requires direct contact with the ocular surface, which may cause patient discomfort. Furthermore, IVCM is not widely available at all institutions, and imaging requires a skilled operator. Additionally, while IVCM can reliably differentiate normal tissue from abnormal tissue, it has poor sensitivity and specificity when attempting to distinguish benign and malignant lesions [140]. As such, IVCM cannot replace histopathologic evaluation in the assessment of ocular surface lesions. Lastly, operators cannot reliably identify landmarks in the tissue they are imaging, so it is difficult to determine the precise location that is being imaged and not feasible to repeatedly image the same location over time.

### 5.4. Recent Advances

Notably, AI has recently been applied to assist with interpretation of IVCM images. In a recent study using 2774 IVCM images, three neural networks were trained to differentiate OSSN from healthy tissue and other ocular surface lesions [142]. When tasked to differentiate OSSN from healthy tissue, the models achieved accuracies of 97–99% and F_1_ scores (a measure of predictive performance that represents the harmonic mean of test precision and recall) of 92–99% [142]. When differentiating OSSN from non-OSSN pathologies, accuracies ranged from 92 to 100% and F_1_ scores ranged from 90 to 100% [142]. While these preliminary results are promising, further research is necessary to apply these AI algorithms to the non-binary classification tasks required in clinical practice. Additionally, there remains a need for development of novel applications of IVCM for ocular surface lesions.

### 5.5. Summary

IVCM is a contact imaging modality that provides ultra-high-resolution, en face images of the ocular surface at the cellular level. While it has been applied to the investigation of various ocular surface lesions, its clinical utility remains limited due to limited availability and requiring a skilled operator. Consequently, IVCM is not routinely used in clinical practice for ocular surface lesions.

## 6. Discussion

The evaluation and diagnosis of ocular surface lesions may present a challenge, as a wide spectrum of benign and malignant pathologies can affect the cornea and conjunctiva, and there is frequent overlap in clinical presentation. Ocular surface imaging modalities such as AS-OCT and UBM are indispensable ancillary tests that regularly supplement the clinical examination and facilitate the diagnosis, management, and monitoring of these lesions. Conversely, OCTA and IVCM are rarely used in the evaluation of ocular surface lesions clinically but may have future potential with increased knowledge.

While each imaging modality can be valuable in its own way (Table 1), multiple imaging techniques may be required to fully assess an ocular surface lesion. For example, evaluation of CM may require both AS-OCT and UBM. In this case, AS-OCT excels in determining if the lesion is epithelial or subepithelial and provides intratumoral detail, whereas UBM excels in identifying the posterior tumor margin and detecting intraocular invasion. Notably, anterior segment imaging cannot entirely supplant histopathologic analysis, and biopsy is often still required for definitive diagnosis of many ocular surface lesions. Important recent advancements in ocular surface imaging include the application of AI to the interpretation of AS-OCT, preoperative and intraoperative use of AS-OCT, the development of 3D UBM, and expanded applications of each modality to a variety of ocular surface lesions. Continued research is required to validate these emerging applications, improve imaging speed and resolution, and establish standardized imaging protocols for the evaluation of ocular surface lesions.

## Figures and Tables

**Table 1:** Comparison of ocular surface imaging modalities.

	AS-OCT	OCTA	UBM	IVCM
**Principle**	Low-coherence interfero35.	Motion contrast detection of blood flow	Acoustic reflection of high-frequency ultrasound waves (35–100 MHz)	Diffraction limited point excitation and signal detection
**Image resolution**	HR: 5–7 μm UHR: 1–5 μm	5 μm (axial) resolution of 3 × 3 mm^2^ area	At 50 MHz, ~25 μm (axial) by ~50 μm (lateral) to a depth of ~5 mm	0.2–4.0 μm (lateral) by 0.6–4.0 μm (axial) to a depth of ~130 μm
**Strengths**	• Non-contact • High resolution • Rapid acquisition • Precise measurements • Accurate differentiation of tissue layers • Morphologic cross section	• Non-contact • Rapid acquisition • Contrast dyes are not required	• Excellent depth of penetration • Real-time images • Capable of imaging thick/heavily pigmented lesions	Allows cellular level visualization
**Limitations**	• Posterior shadowing in thick/heavily pigmented lesions	• Motion and projection artifacts • Highly operator-dependent • Cannot distinguish arteries from veins • Poor penetration in opaque tissue	• Requires direct contact with eye • Relatively poor image resolution • Highly operator-dependent	• Current devices requires direct contact with eye • Small, single-plane field of view • Time-consuming image acquisition • Requires skilled operator and expert interpretation • Challenging to identify scan location

AS-OCT = Anterior segment optical coherence tomography; HR = high-resolution; IVCM = in vivo confocal microscopy; OCTA = optical coherence tomography angiography; UBM = ultrasound biomicroscopy; UHR = ultra-high-resolution.

## Data Availability

No new data were created or analyzed in this study. Data sharing is not applicable to this article.
